# High CO_2_ adaptation mechanisms revealed in the miR156-regulated flowering time pathway

**DOI:** 10.1371/journal.pcbi.1011738

**Published:** 2023-12-20

**Authors:** Kun Zhang, Erkang Wang, Qiong Alison Liu, Jin Wang

**Affiliations:** 1 School of Applied Chemistry and Engineering, University of Science and Technology of China, Hefei, Anhui, P. R. China; 2 State Key Laboratory of Electroanalytical Chemistry, Changchun Institute of Applied Chemistry, Chinese Academy of Sciences, Changchun, Jilin, P.R. China; 3 IvyGen LLC, Wilmington, Delaware, United States of America; 4 Department of Chemistry and of Physics, Stony Brook University, Stony Brook, New York, United States of America; University of California, Riverside, UNITED STATES

## Abstract

Elevated CO_2_ concentrations have been observed to accelerate flowering time in Arabidopsis through the action of a highly conserved regulatory network controlled by miR156 and miR172. However, the network’s robustness to the impact of increasing CO_2_ concentrations on flowering time remains poorly understood. In this study, we investigate this question by conducting a comprehensive analysis of the global landscape of network dynamics, including quantifying the probabilities associated with juvenile and flowering states and assessing the speed of the transition between them. Our findings reveal that a CO_2_ concentration range of 400–800ppm only mildly advances flowering time, contrasting with the dramatic changes from 200 to 300ppm. Notably, the feedback regulation of miR156 by squamosal promoter binding protein-like proteins (SPLs) plays a substantial role in mitigating the effects of increasing CO_2_ on flowering time. Intriguingly, we consistently observe a correlation between delayed flowering time and increased variance in flowering time, and vice versa, suggesting that this might be an intrinsic adaptation mechanism embedded within the network. To gain a deeper understanding of this network’s dynamics, we identified the sensitive features within the feedback loops of miR156 SPLs and miR172—APETALA2 family proteins (AP2s), with the latter proving to be the most sensitive. Strikingly, our study underscores the indispensability of all feedback regulations in maintaining both juvenile and adult states as well as the transition time between them. Together, our research provides the first physical basis in plant species, aiding in the elucidation of novel regulatory mechanisms and the robustness of the miRNAs-regulated network in response to increasing CO_2_, therefore influencing the control of flowering time. Moreover, this study provides a promising strategy for engineering plant flowering time to enhance their adaptation and resilience.

## Author summary

This study investigates the impact of elevated atmospheric CO_2_ levels on the flowering time of Arabidopsis plants based on the underlying genetic regulatory network. While genetic insights into this pathway have been gained, it remains unclear how resilient this network is to increased CO_2_ levels and what its adaptive mechanisms is. We conducted a dynamical modeling and associated analysis on the network, exploring how CO_2_ concentrations influence flowering time. We employed the landscape and flux theory to quantify the global landscape for network dynamics, estimate the likelihood of different reproductive states, and assess transition speeds between them. By introducing "mutations" into the system, we identified the basin locations and depths of the landscape as well as the key elements in network. The study uncovered that the miR156—SPLs feedback circuit is responsible for stabilizing the pathway and mitigating the effects of elevated CO_2_, while the miR172—AP2s feedback circuit is well-stabilized but highly sensitive. This feedback mechanism in plant species offers valuable insights into how plants adapt to environmental challenges. The current study provides a foundation of understanding how miRNA feedback circuits respond to increased CO_2_ levels, suggesting future strategies for genetically engineering plant flowering to enhance their resilience.

## Introduction

Non-coding microRNAs (miRNAs) function as important regulators of gene expressions in nearly all eukaryotes. Their primary role involves gene silencing through the sequence-specific cleavage of messenger RNAs (mRNAs) or by repressing mRNA translation [1,2]. Studies have revealed that miRNAs can enhance phenotypic canalization by buffering fluctuations in their mRNA targets, which often encode transcription factors that play pivotal roles in developmental pathways [3]. These effects are frequently orchestrated through feedback loops regulated by miRNAs. It is widely acknowledged that miRNAs are highly responsive to environmental changes, exhibiting dynamic fluctuations in expression levels. These fluctuation, in turn, modulate the abundance of their targeted mRNAs and proteins [3,4].

**Fig 1:**
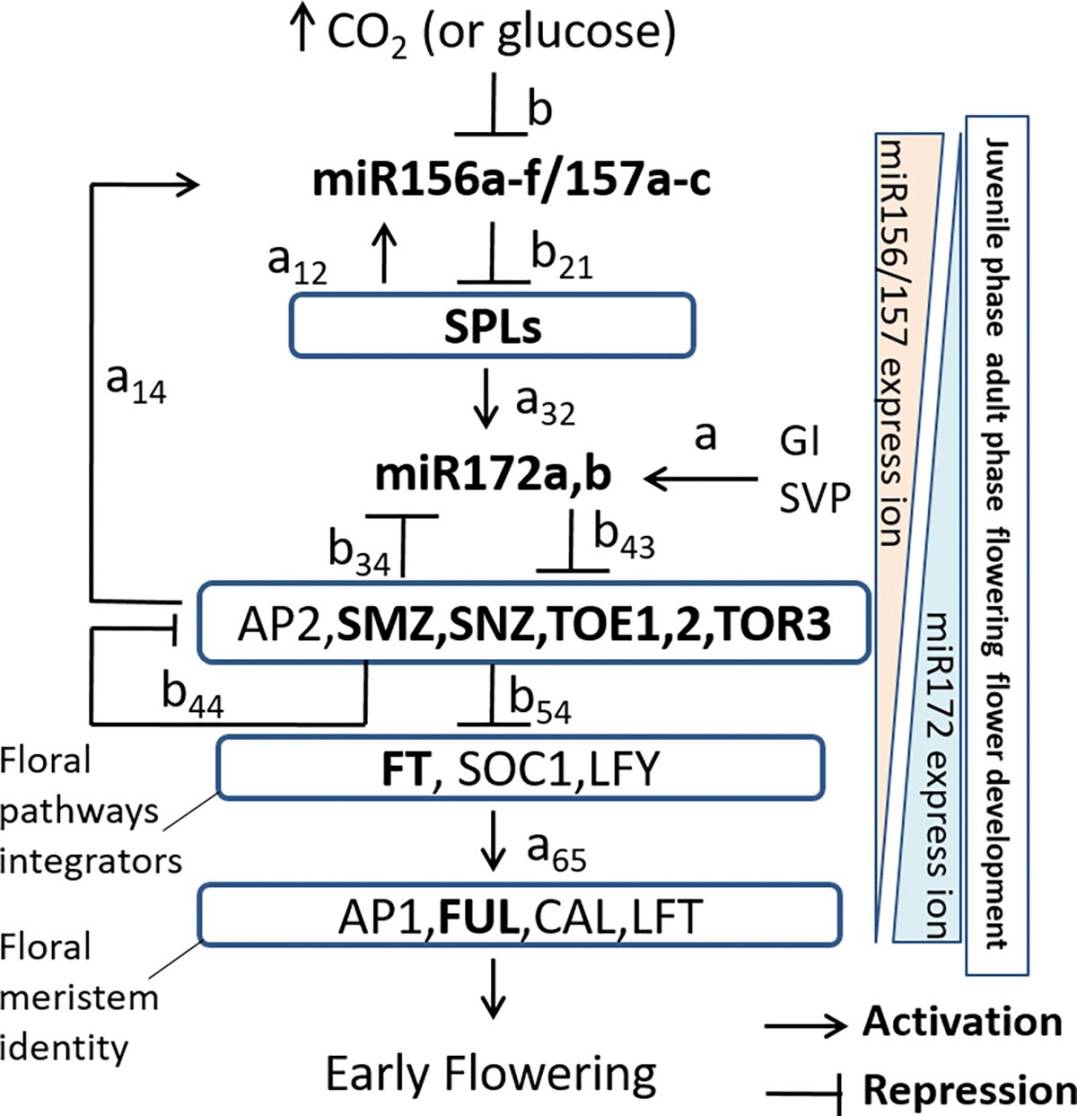
The effects of elevated [CO_2_] (or glucose) on the miR156/157-regulated flowering time regulatory network. Bold highlights genes whose expressions can be affected by elevated [CO_2_]. Within the boxes are all the genes that are known in regulating developmental flowering time. GI and SVP were not affected in expression by elevated [CO_2_]. The relationship between CO_2_ and glucose is represented by the photosynthesis reaction: 6CO_2_ + 6H_2_O →C_6_H_12_O_6_ + 6O_2_.

In *Arabidopsis*, genetic studies have demonstrated the central role of miR156/157 and miR172 in orchestrating the transition from the vegetative juvenile to adult stages transition and in regulating flowering time. These processes are primarily governed by their sequential control of two families of transcription factors: SPLs and AP2s [5,6] (Fig 1). It is worth noting that miR156 and miR172, being miRNAs of ancient origins, exhibit a remarkably conserved role in controlling reproduction timing across diverse plant species [7,8].

Specifically, miR156 exerts its regulatory control by targeting and suppressing the expressions of multiple members of the SPL family [6,9,10,11,12,13,14,15]. Certain SPLs can, in turn, promote the transcription of miR156 precursor gene [6], establishing a feedback circuit in the miR156—SPLs interaction. Throughout *Arabidopsis* development, miR156 levels are highest in young seedlings and gradually decrease over several weeks as the plant transitions into the flowering stage [10]. A reduction in miR156 level leads to the upregulation of its target transcription factor, SPL9, subsequently increasing the expression of miR172 precursor gene and elevating miR172 level [6,10].

Genetic studies have unveiled that miR172 plays an pivotal role in advancing flowering time by suppressing the expression of its target transcription factors belonging to the AP2 family, such as AP2 and AP2-like TOE1, TOE2, TOE3, SMZ and SNZ, primarily through translational suppression [6,10]. This downregulation of the AP2 family alleviates their transcriptional inhibition of floral pathway integrators and floral meristem identity genes, ultimately promoting adult epidermal identity and flowering [6,10]. Additionally, AP2 can transcriptionally suppress the expression of miR172, creating a feedback loop within the miR172—AP2 interaction [16]. The interplay among AP2 family members also involve negative regulation, further adding complexity to the miR172-AP2 feedback loop [16,17].

MiR156 and miR172 exhibit significant responsiveness to various environmental stressors including heat, cold, salt, and drought [18,19]. In *Arabidopsis*, the miR156 transcription network displays a specific response to elevated CO_2_ levels, where all components in the network undergo correlated alterations, ultimately leading to an early onset of flowering [20] (Fig 1). This phenomenon suggests that CO_2_ may influence this network through its photosynthetic products, glucose and sucrose, which can downregulate miR156 by inhibiting the expression of its precursor genes, *MIR156A* and *MIR156C* [21,22]. Importantly, HEXOKINASE1 (HXK1), a regulator of glucose signaling, is indispensable for mediating this effect [21] and a glucose metabolite trehalose-6-phosphate (T6P) also plays a role in this process [23].

This miR156-regulated network is highly complex, characterized by a multitude of regulatory features. These include miRNAs-mediated suppression of gene expression, both transcriptional activation and suppression of gene expression, the presence of redundancy within transcription factor family, and the presence of multiple positive and negative feedback loops (Fig 1). Previous genetic studies have made significant progress in unraveling the regulatory mechanisms and dynamics of this network. Notably, these studies have provided insights into the expression levels of both miRNAs and transcription factors across various developmental stages, along with critical expression thresholds that initiate the onset of flowering [24].

However, genetic approach has limitations in capturing the overall dynamic changes within the network, such as the speed in which these changes occur. Additionally, it is challenging to pinpoint which specific genes or gene families, as well as regulatory mechanisms, wield the most influence in shaping this critical transition from vegetative to reproductive phases and in determining flowering time. Answering these questions is of crucial importance for devising strategies to prevent and address aberrations in flowering time.

In this study, we embarked on a quantitative exploration of this developmental network, aiming to understand the influence of CO_2_ concentration on flowering time, taking into account of the roles played by miR156/157 and miR172 and all other components and regulations within this complex network. So far, there have not been any reported studies using the similar approach investigating this network. Our approach allows us to unveil the global landscape of the gene regulatory network dynamics and decipher the probabilities of juvenile and flowering states as well as the speed of the transition between them. We also introduced “mutations” to pinpoint the locations and depths of developmental states within the network influenced by specific genes and their regulatory interactions. Our findings indicate that elevating CO_2_ levels from 400 to 800 ppm leads to a modest advancement in flowering time. In simpler terms, this suggests that the response curve for flowering time exhibits a relatively shallow or a gentle change in response to alternations in CO_2_ concentrations. Notably, miR156/157 detects the increasing CO_2_ signal, but its impact on flowering time is significantly tempered by the feedback regulation mediated by SPLs. Intriguingly, we observed a consistent correlation between delayed flowering time and increased variability in flowering time, and vice versa. This correlation hints at the presence of an intrinsic adaptive mechanism embedded in the network. Furthermore, our investigation revealed that the regulatory mechanisms within the miR172-AP2 family and miR156-SPLs modules are the most responsive to changes in elevated CO_2_, exerting significant influence on flowering time. Our study also sheds light on the evolutionary importance of these feedback regulations within the network, as they are vital for maintaining the states of juvenile and flowering, as well as regulating the transition between them. Our study not only unveil novel functions of the regulatory mechanisms within the network but also offers potential strategies for modifying the trait of plant flowering time in response to the increasing atmospheric CO_2_.

## Material and method

### Quantifying the miR156/157 regulated transcription network

Our quantification method was inspired by the global picture of Waddington landscape for development and differentiation [25], demonstrating that the complex system dynamics can be globally determined by two driving forces [26,27,28], the probability landscape of the system, and the associated curl probability flux. The former quantifies the probability of each state while the latter quantifies the flow between the states.

The miR156/157 regulatory network determining the juvenile-to-adult transition and flowering time in *Arabidopsis* is shown in Fig 1. The regulatory network shows that elevated CO_2_ concentration might affect the whole network by its photosynthetic sugar products, glucose and sucrose. Elevated CO_2_ hastens the decrease in miR156 and its homologue, miR157 expression, and the correlated expression changes in the downstream components. The SPLs were increased in expression due to the suppression of miR156/157, thus directly upregulates the transcription of miR172, which further suppress AP2 and AP2-like transcription factors. TOE2, SNZ, SMZ, TOE1, and TOE3 and AP2 also negatively regulate each other. Moreover, miR172 expression can be also transcriptionally regulated by GI and SVP. Meanwhile, the SPLs feedback-regulate miR156 precursor transcription and AP2 family negatively regulates the expression of miR172 and positively regulates the miR156/157. In summary, a schematic diagram indicates the decline of miR156/157 and the rise of miR172 expression from the juvenile to the flowering development during plant development on the effects of elevated CO_2_.

The miRNA156/157 network is highly conserved among plant species. The regulatory wiring structure of this network has been extensively studied genetically shown in the previous literature [5,6]. We construct the deterministic part of the driving force **F**(**x**) and the associated six dynamical equations of the model by analyzing the regulation relationship among transcription factors and microRNAs in the flowering regulatory network.

A set of six ordinary differential equations, based on this gene regulatory wiring diagram, were constructed representing the miR156/157 regulatory network dynamics as follows,

$$\begin{array}{l} \frac{dX_{1}}{dt}=\frac{a_{12}X_{2}^{n}}{S^{n}+X_{2}^{n}}+\frac{a_{14}X_{4}^{n}}{S^{n}+X_{4}^{n}}+bm(1-b+\frac{bS^{n}}{S^{n}+{\left(c[CO_{2}]\right)}^{n}})-kX_{1}\\ \frac{dX_{2}}{dt}=bm_{21}(1-b_{21}+\frac{b_{21}S^{n}}{S^{n}+X_{1}^{n}})-kX_{2}\\ \frac{dX_{3}}{dt}=\frac{a_{32}X_{2}^{n}}{S^{n}+X_{2}^{n}}+bm_{34}(1-b_{34}+\frac{b_{34}S^{n}}{S^{n}+X_{4}^{n}})+\frac{a{\left[SVP\right]}^{n}}{S^{n}+{\left[SVP\right]}^{n}}-kX_{3}\\ \frac{dX_{4}}{dt}=bm_{44}(1-b_{44}+\frac{b_{44}S^{n}}{S^{n}+X_{4}^{n}})+bm_{43}(1-b_{43}+\frac{b_{43}S^{n}}{S^{n}+X_{3}^{n}})-kX_{4}\\ \frac{dX_{5}}{dt}=bm_{54}(1-b_{54}+\frac{b_{54}S^{n}}{S^{n}+X_{4}^{n}})-kX_{5}\\ \frac{dX_{6}}{dt}=\frac{a_{65}X_{5}^{n}}{S^{n}+X_{5}^{n}}-kX_{6} \end{array}$$

Here, X_i_ (i = 1, 2…6) represents the expression or concentration of miR156/157, SPL family, miR172, AP2 family, FT, and AP1 family, respectively. a_ij_ is the activation strength parameter from gene *j* to gene *i*. b_ij_ is the repression strength parameter from gene j to gene i. bm_ij_ is the maximum rate of the repression regulated parameter from gene j to gene i. k is the degradation rate constant. *n* is Hill coefficient. *S* is the Hill constant, or the threshold represented by the half-maximum effective concentration values. (Table A in S1 Text) The mathematical form of the deterministic part of model, or the nonlinear interactions, is still a semi-quantitative and empirical. It has been widely used in different regulatory network models because it can represent the relationships between the various components of the network. However, many of the parameters in the model are still empirically estimated, which means that they are not known with certainty [29]. Obtaining a precise parameter estimate would require gathering additional experimental data with greater detail. We can obtain a model of multiple stable states by using the regulatory rules and the empirical parameter estimations for the Hill equation. The unity parameters were chosen to simplify the interactions while being consistent with empirical findings. This avoids the almost impossible parameter search space and allows us to focus on the study of the dynamics of the network with the same wiring topology. We expect similar dynamical behaviors or scaling in networks with the same wiring topology but different parameters.

Transcription factors and microRNAs regulate genes in different ways. Transcription factors bind to DNA sequences within the regulatory regions of target genes, while miRNAs bind to mRNAs and either degrade them or inhibit their translation into proteins. In this study, we used Hill functions to model the regulation of genes by transcription factors and miRNAs. Hill functions are commonly used in biochemistry, pharmacology, molecular biology, and systems biology to describe processes where the response to a molecular concentration is not linear. The Hill equation describes a sigmoidal curve, which means that the response increases sharply near a threshold value, representing the transition from a low to a high response state. Both the regulation of transcription factors and miRNA-mediated feedback loop can behave as switches [30,31]. We approximately characterize the feature with the Hill functions.

The intrinsic statistical fluctuation due to both a finite number of molecules inside a cell and external fluctuations of cellular environment can significantly impact on the network dynamics. We thus took stochastic fluctuations into consideration in describing the dynamics of flowering time. Therefore, the dynamics of the network was formulated as the stochastic differential equations with the noise, d**x**/dt = **F**(**x**)+η, where **x** is the expression level of a gene and **F**(**x**) is a vector representing the driving force of the system. η represents Gaussian white noise with zero mean and its autocorrelation function is given as <η(0)η(t)> = 2**D**δ(t). **D** is the diffusion coefficient, which characterizes the intensity of the intrinsic and cellular environmental fluctuations. The stochastic process is similar to the Brownian dynamics.

Since the time trajectory of the expression level or concentration dynamics is not deterministic due to the stochastic fluctuations, the probability distribution is more appropriate for a quantitative description. The evolution of the probability patterns is dictated by the probability evolution equation, which is often linear in the probability P itself. For example, the Fokker-Planck equation and the master equation are both typically linear in the probability. We thus did statistical analysis, calculating the probability distribution at the steady state through simulating the time evolution of underlying stochastic dynamics. On the other hand, the probability evolution follows the diffusion equations for continuity [32]. The equation is also called Fokker-Planck equation that can be written in the form of probability conservation: ∂P(x,t)/∂t=−∇⋅(F(x)P(x,t)−D∇P(x,t)), where **F**(**x**) is mentioned above driving force of system and **D** is diffusion coefficient. At the long-time limit (steady state), the left side of the Fokker-Planck equation ∂P(x,t)/∂t is equal to zero. Therefore, the steady state probability distribution P_ss_(**x**) (P_ss_ = P(x,t→∞)) satisfied the following equation $\nabla \cdot (F(x)P_{ss}(x)-D\nabla P_{ss}(x))=0$. The steady state probability P_ss_ characterizes the weight of each possible state **x**. This forms a probability landscape characterizing not only the weight but also the possible global connections from one state to another. The state with higher weight corresponds to the more probable state of appearance. U(**x**) is defined as the population landscape related to the steady state probability U = -lnP_ss_. We can solve the steady state probability distribution, P_ss_ from the Fokker-Planck equation and quantify the population landscape, U = -lnP_ss_. The population landscape quantifies the basins as more probable functional states and the possible global connections between the basins based on the state weights involved. The right-hand side F(x) of the stochastic differential equations is the total deterministic driving force of the system dynamics. In general, this force can be decomposed into two terms: the gradient of the population potential landscape, and a rotational flux force. When a complex system satisfies the detailed balance condition, the system will reach an equilibrium state after a long time evolution, and the rotational flux force will be zero. However, general open systems do not obey this condition. Therefore, the dynamics of a complex system is determined by two driving forces, which are analogous to a charged particle moving under the electric and magnetic fields, descending down along the electric field gradient and spiraling due to the rotational Lorentz force exerted from a magnetic field. While the landscape component determines the overall direction of the system’s evolution, the flux component can make the dominant path from the flowering state to the juvenile state different from that from the juvenile state to the flowering state. The Mean First Passage Time (MFPT) is an important concept in the analysis of stochastic processes. It is the expected time it takes for a stochastic process to reach a certain state or position for the first time, starting from a given initial state or position. The MFPT can be used to measure the expected duration for a process to switch from one state to another, such as the cell fate decision-making process. In the flowering model characterized by the Langevin equation or Fokker-Planck equation, the MFPT can be used to predict how long it takes for the system to reach a flowering state, starting from the juvenile state. The calculation of the MFPT can be complex, depending on the specific stochastic process being studied. It often involves solving differential equations or using probabilistic methods to analyze the behavior of the process over time. In this study, we investigated the MFPT by simulating a large number of trajectories using stochastic differential equations. We started each trajectory from the juvenile state and recorded the time at which each trajectory first reached the flowering state. We then performed a statistical analysis of the collected data to obtain the mean value and probability distribution of the first passage time.

## Results

### Identification of two steady states in the network landscape

**Fig 2:**
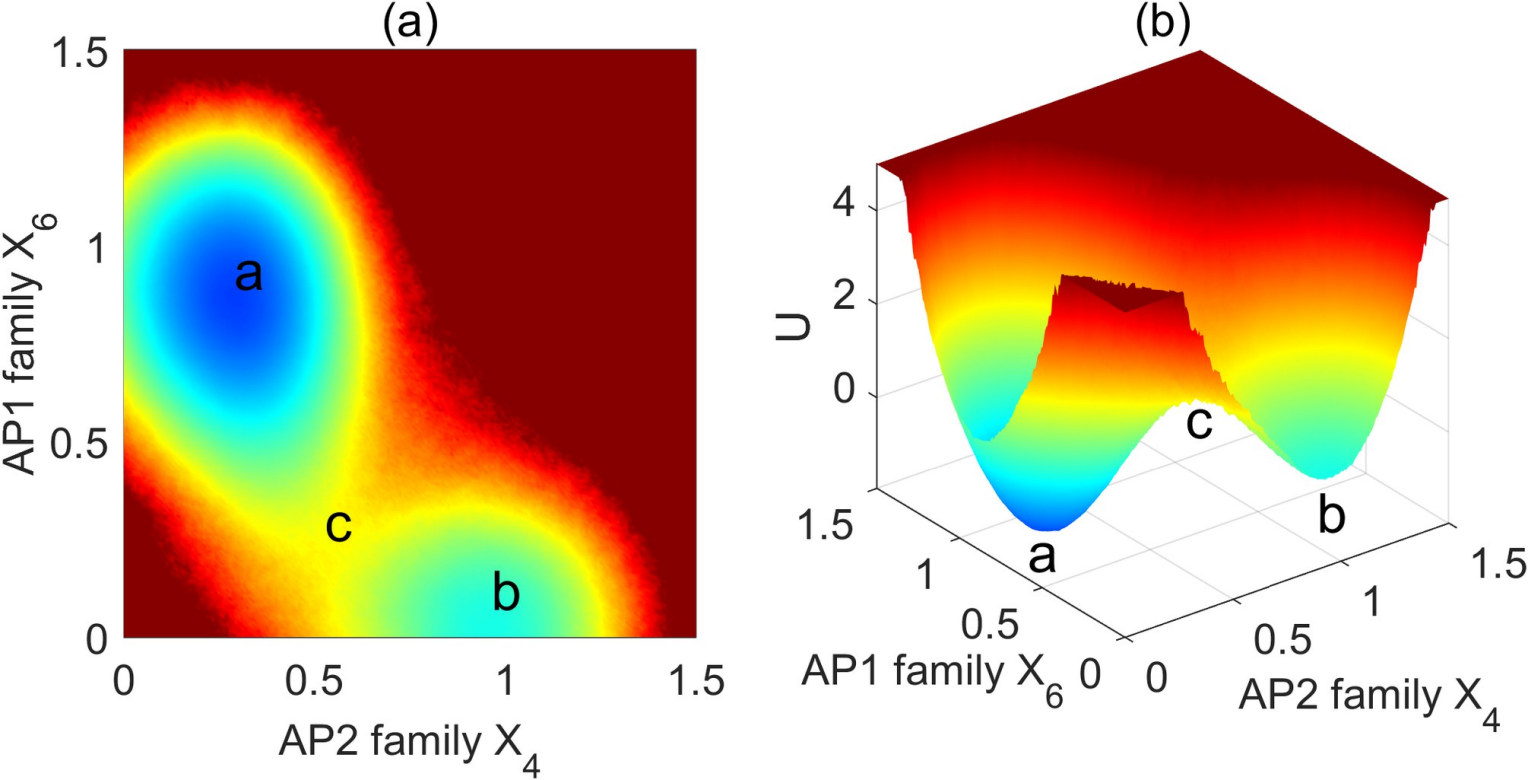
(a) The two dimensional landscape of the flowering network. (b) The three dimensional landscape of the flowering network. (The state “a” denotes the flowering state; the state “b” denotes the juvenile state; the state “c” is the saddle point of the landscape).

Through our quantitative investigations, we have identified two steady states within the network landscape: the juvenile state and the flowering state. In this context, the AP2 family and the floral meristem identity genes exhibit characteristics indicative of steady state in the landscape. The probability distribution of these steady-states quantifies the likelihood of each expression state, with the population landscape U being directly associated with the steady state probability, calculated as U = -lnP_ss_. The topography of the underlying population landscape can be visualized through the depiction of the basins and barriers. For the population landscape of the flowering system, each state—whether it occurs before or after flowering—has a different probability. The basins or valleys within the landscape represent the most probable functional states of the system. To provide a visual representation of this population landscape, we present both two- and three-dimensional projections (Fig 2A and 2B). We derived the landscape by analyzing the two-dimensional marginal probability distribution of the AP2 family (X_4_) and floral meristem controlling genes (X_6_). Within this landscape, we identified two prominent basins representing high-probability functional states. These basins correspond to the AP2 family (X_4_) and floral meristem controlling genes (X_6_), symbolizing the juvenile state and the flowering state in *Arabidopsis* development. The “a” basin exhibits significantly greater depth, compared to the “b” basin (Fig 2A and 2B), suggesting the transition direction from the vegetative state “b” to the flowering state, “a”. In fact, the flowering and juvenile states of a plant are jointly determined by six dynamic variables. To visualize the landscape of the flowering network, we can project it onto the landscape of two variables. This means that we can average out the effects of the other four variables for a particular chosen pair of variables. This allows us to see the corresponding basin of two stable states on the projected landscape. We can also project the landscape onto other variables. The two stable states can still be obtained by solving the fixed points of the associated six-dimensional dynamical differential equations dx/dt = F(x).

### Elevated CO_2_ concentrations, ranging from 400 to 800ppm induce a slight acceleration in flowering time

To understand the impact of elevated CO_2_ on *Arabidopsis* flowering time, we conducted an analysis using the mean first passage time (MFPT) as a measure to quantify the flowering time across a range of CO_2_ concentrations, spanning from near-zero levels up to 800ppm. The First Passage Time (FPT), being a kinetic time parameter, is subject to fluctuations. The statistical property or distribution of the kinetic time can be characterized. This distribution, referred to as the First Passage Time Distribution P(t), provides insights into the mean transition time between different states, which is termed MFPT. In our study, we employed MPFT to quantify the transition from the juvenile state “b” to the adult flowering state, “a”, effectively representing the flowering time. By quantifying the landscape, as depicted in Fig 2, we observed that the transition of the system from the juvenile state to the adult flowering state must overcome a barrier. Consequently, MPFT is expected to be correlated with the height of this barrier, situated between the saddle point “c” and the juvenile state “b”.

**Fig 3:**
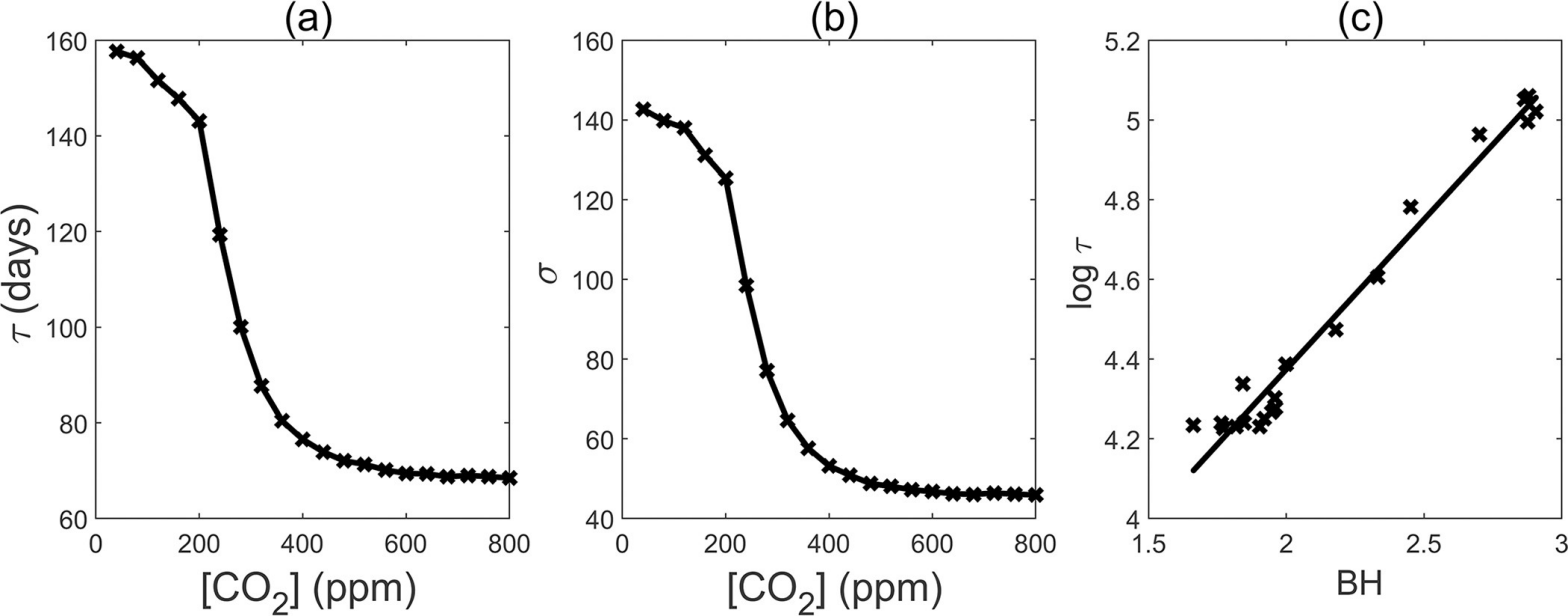
(a) Mean first passage time (MFPT, Unit: days) with different [CO_2_](Unit: ppm). (b) Standard deviation with different [CO_2_]. (c) Logarithm of MFPT versus barrier height (BH = U_c_-U_b_) in different [CO_2_].

We observed a sharp decline in MFPT as CO_2_ concentrations increased from 200ppm to 300ppm and eventually reaches to a plateau around 400ppm (Fig 3A). This trend was mirrored by a corresponding reduction in the standard deviation of the first passage time (Fig 3B). These findings indicate that increasing CO_2_ can greatly expedite flowering between 200ppm and 400ppm, but exhibit diminishing effects beyond the 400ppm threshold. Furthermore, the variation of flowering time demonstrated a correlated decrease. Our model was fine-toned based on experiment results, showing that *Arabidopsis* plants exposed to a 810 ppm CO_2_ concentration flowered approximately 10% earlier than those cultivated at 430 ppm [21]. We employed days as our time unit. We believe that this is a reasonable timescale unit because it is consistent with the simulation and experimental data on flowering time. Typically, under natural conditions, *Arabidopsis* flowering occurs approximately 70–80 days after germination [20].

We also plotted the relationship between the MFPT and the barrier height from the saddle point “c” to the juvenile state “b” across various concentrations of CO_2_ (Figs 2B and 3C). Our analysis revealed a positive correlation between the logarithm of MFPT and the barrier height. Hence, as the landscape barrier height increases, it becomes increasingly difficult to overcome, resulting in a prolonged transition time between states.

In our modeling framework, flowering is controlled mainly by its own flowering regulatory network at different developmental stages. However, environmental conditions can also influence flowering by affecting the external noise or the changes in regulating the miRNA. Indeed, flowering can be reversed in our model, but the timescale is much longer than the normal flowering. This is because the barrier height of normal flowering (Uc-Ub in Fig 2) is shallower than the barrier height of the reversed process (Uc-Ua in Fig 2). The MFPT is positively correlated with the exponent of the barrier height. Therefore, the flowering time is much shorter than the time of the reversed process. The much longer timescale indicates that the reversed process won’t happen in reality. It is important to note that the flowering time is determined by both the barrier height and the noise level. The barrier height is mostly determined by the network regulations.

### The miR156 regulation of SPLs is both sensitive and effective in modulating flowering time

**Fig 4:**
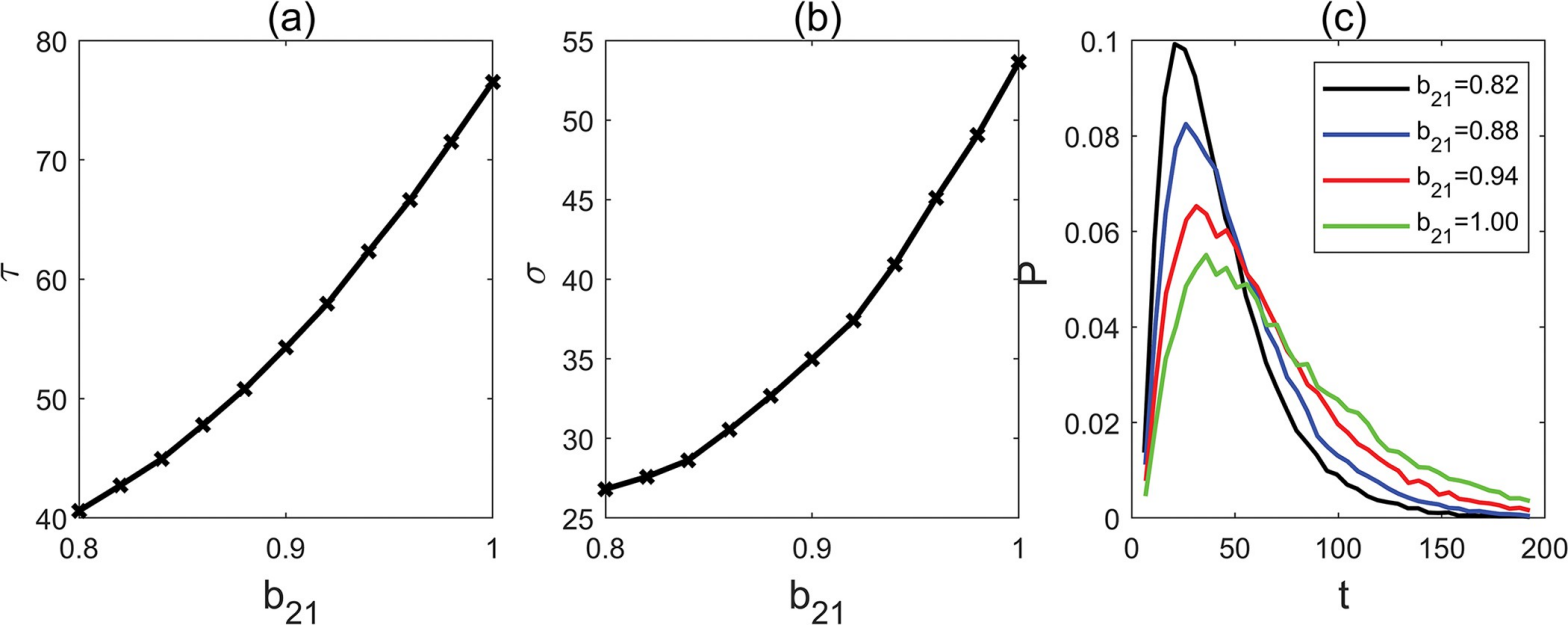
(a) Mean first passage time with different parameter b_21_. (b) Standard deviation with different parameter b_21_. (c) The distribution of first passage time with different parameter b_21_.

To understand how miR156/157 expression levels affect flowering time, we conducted an analysis of MFPT by manipulating the parameter b_21_, which represents the inhibitory strength of miR156/157 on SPLs transcription factors (Fig 4). In our study, a value of “1” was assigned to represent the highest level of inhibition, reflecting a natural condition. Our results demonstrate a consistent reduction in MFPT, when the parameter b_21_ is moderately downregulated from 1 to 0.8. However, below the threshold 0.8, both the basin “b” and the hurdle vanish. This denotes that only a small decrease in the parameter “b_21_”, representing the regulatory strength from miR156/157 on SPLs, can lead to a significant advancement in flowering time.

We also found that the FPT distribution narrows substantially with a mild decrease in b_21_ (Fig 4C). This suggests that even a small decrease in the regulatory strength of miR156/157 on SPLs may significantly reduce the range of flowering time.

Thus, our results demonstrate that the miR156 regulation of SPLs is highly sensitive in mediating the effect of elevated levels of CO_2_ on the network, ultimately leading to the transition from the juvenile to adult phase and impacting flowering time.

### The feedback loop that SPLs regulate miR156 significantly reduces the impact of high CO_2_ on flowering time

**Fig 5:**
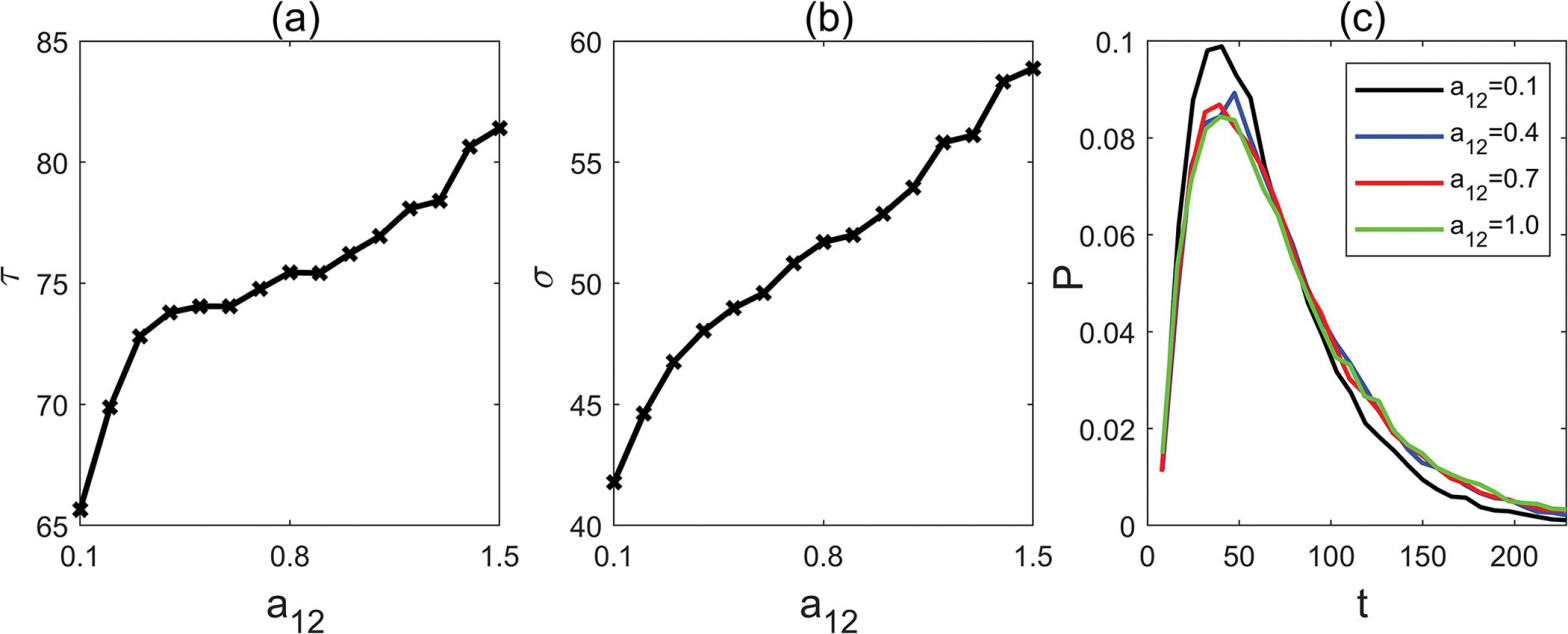
(a) Mean first passage time with different parameter a_12_. (b) Standard deviation with different parameter a_12_. (c) The distribution of first passage time with different parameter a_12_.

It has been previously demonstrated that members of the SPL family can exert transcriptional control over the expressions of miR156 precursor. Based on this knowledge, we conducted quantitative investigation to assess how this feedback mechanism affects flowering time. Our analysis reveals a clear trend: the MFPT increases monotonically as we enhance the parameter a_12_ (Fig 5); a_12_ depicts the strength of SPLs transcriptional regulation of miR156 precursor. This increase in the activation strength, a_12_, led to the increased expression of miR156/157, which, in turn, tends to delay the onset of flowering. Conversely, when a_12_ levels are reduced, miR156/157 expression decreases, resulting in an acceleration of flowering time.

To assess how changes in the parameter a_12_ affects the range of flowering times, we examined the distribution of first passage times with varying a_12_ values (Fig 5C). Our observations indicate that as the activation strength a_12_ decreases, the most probable FPT remains nearly unchanged, and the distribution of FPT only experiences a slight narrowing. Consequently, when a_12_ is increased, both the MFPT and the variance in flowering times do not exhibit significant alteration.

**Fig 6:**
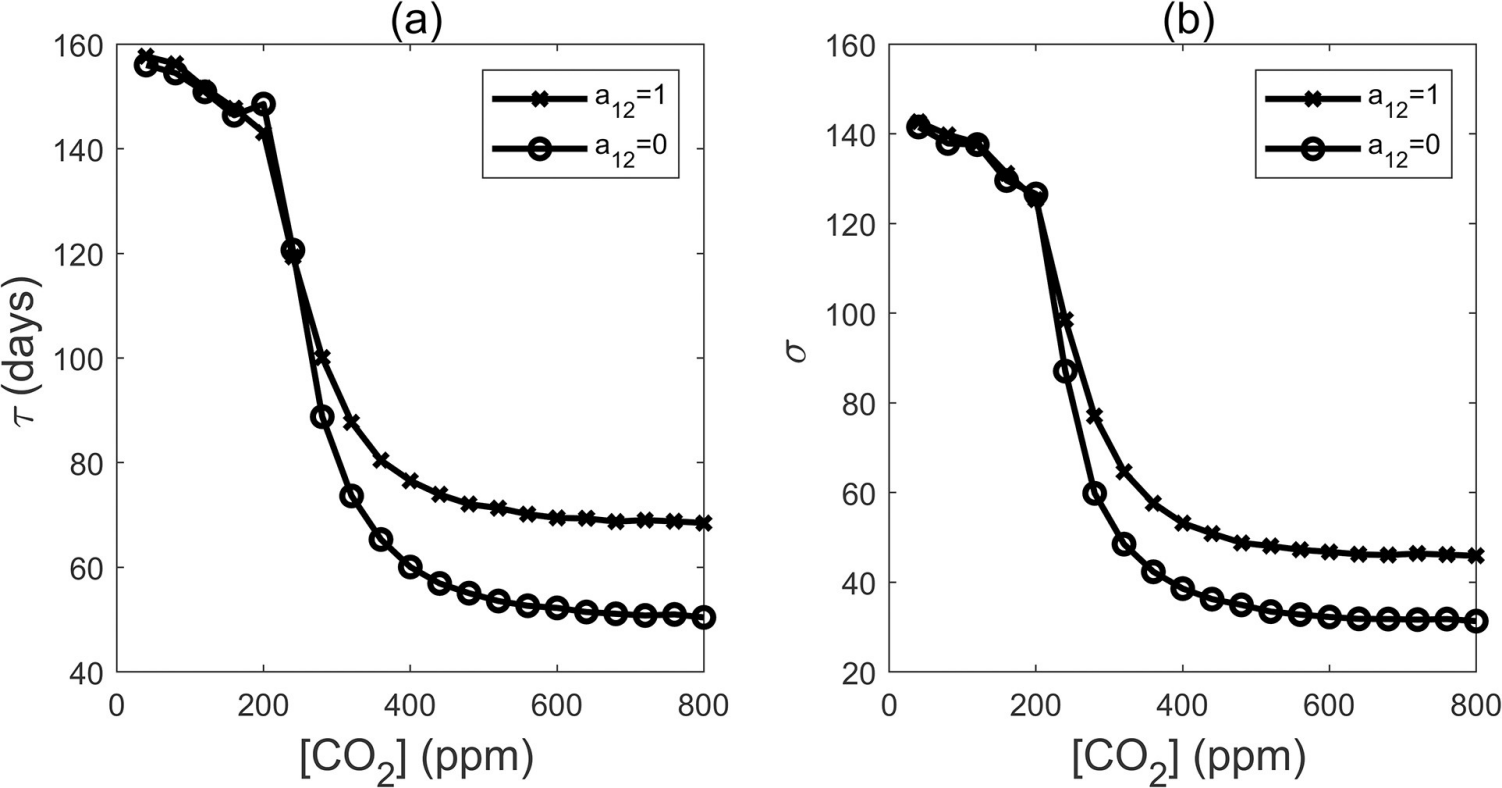
(a) Mean first passage time (MFPT, Unit: days) after removing the feedback regulation of SPLs to miR156/157. (b) Standard deviation with different [CO_2_] after removing the feedback regulation a_12_.

To elucidate the significance of SPL family feedback regulation on miR156 in controlling the effects of elevated CO_2_ levels on plant flowering time, we removed the a_12_ feedback regulation and then calculated the MFPT and its variance (Fig 6). We found that within the range of CO_2_ concentrations spanning from 400 to 800ppm, the mild advancement of flowering time caused by increased CO_2_ levels is disrupted when the feedback regulation is absent. In fact, in the absence of this feedback regulation, flowering time advanced substantially by approximately 20 days. The variance of the flowering time is also reduced significantly. Our results demonstrate that the SPL family’s feedback regulation of miR156 plays a pivotal role in buffering the impact of elevated CO_2_ levels on plant flowering time.

Taken together, our data conclusively demonstrate that changes in miR156 regulation of SPLs drives the shifts of *Arabidopsis* flowering time. Furthermore, the feedback regulation of miR156 by the SPL family plays a pivotal role in curbing the extent of flowering time alteration and its variability.

### The miR172-AP2s module plays a pivotal role in facilitating the transition to flowering time

**Fig 7:**
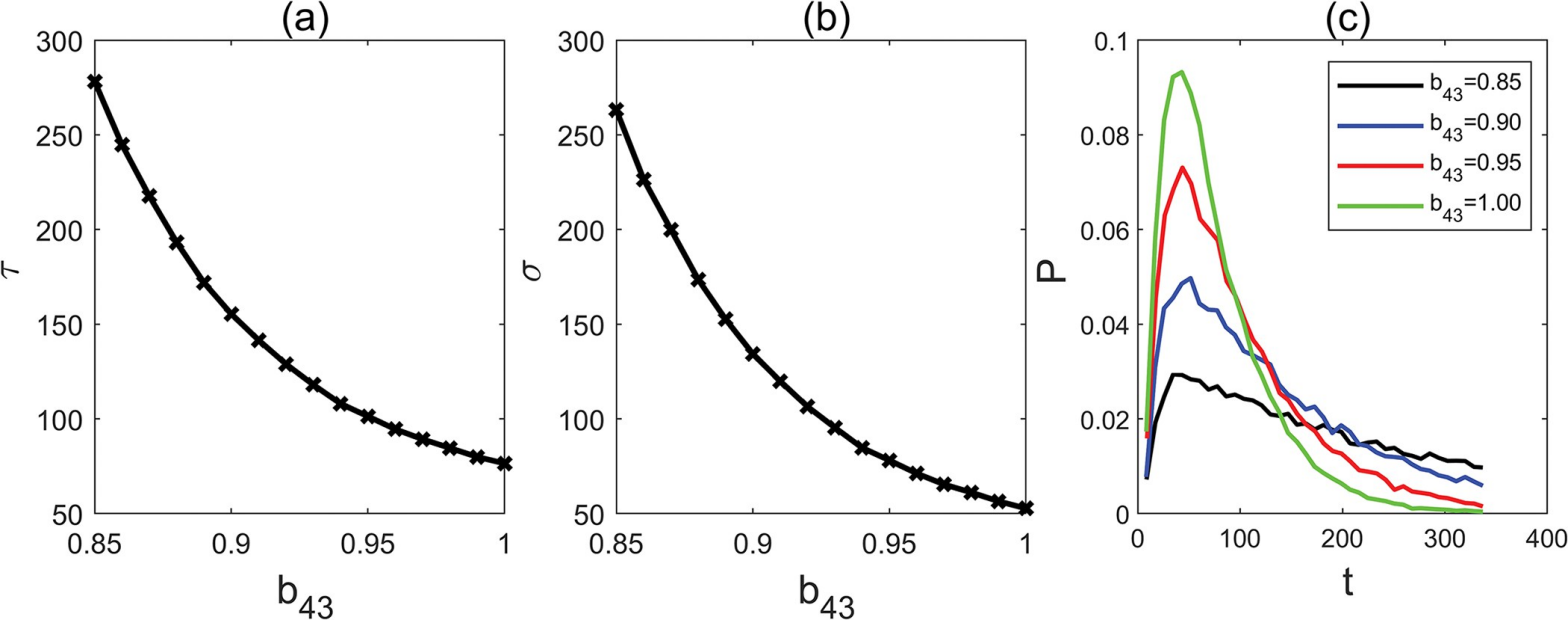
(a) Mean first passage time with different parameter b_43_. (b) Standard deviation with different parameter b_43_. (c) The distribution of first passage time with different parameter b_43_.

It is well-established that miR172 targets AP2 family members mainly by inhibiting their translation, while AP2 family members are known for their role in transcriptionally suppressing the expression of miR172. To understand how the miR172-AP2s module regulates flowering time, we first investigated how the miR172’s suppression of the AP2 family affects flowering time. We accomplished this by examining the MFPT under varying levels of the inhibition-regulated parameter b_43_ (Fig 7A and 7B). Our findings revealed a consistent, monotonic decline in MFPT as the strength of b_43_ increased within the range of 0.85 to 1, with CO_2_ concentration held constant. This suggests that even a small increase in b_43_ can significantly expedite the mean flowering time. Moreover, we observed that the MFPT range narrows considerably with higher b_43_ strength compared to lower strength (with CO_2_ concentration fixed; Fig 7C). On the other hand, the FPT distribution exhibited a reduction in breadth as b_43_ increases (Fig 7B), signifying a decrease in the range of flowering time.

**Fig 8:**
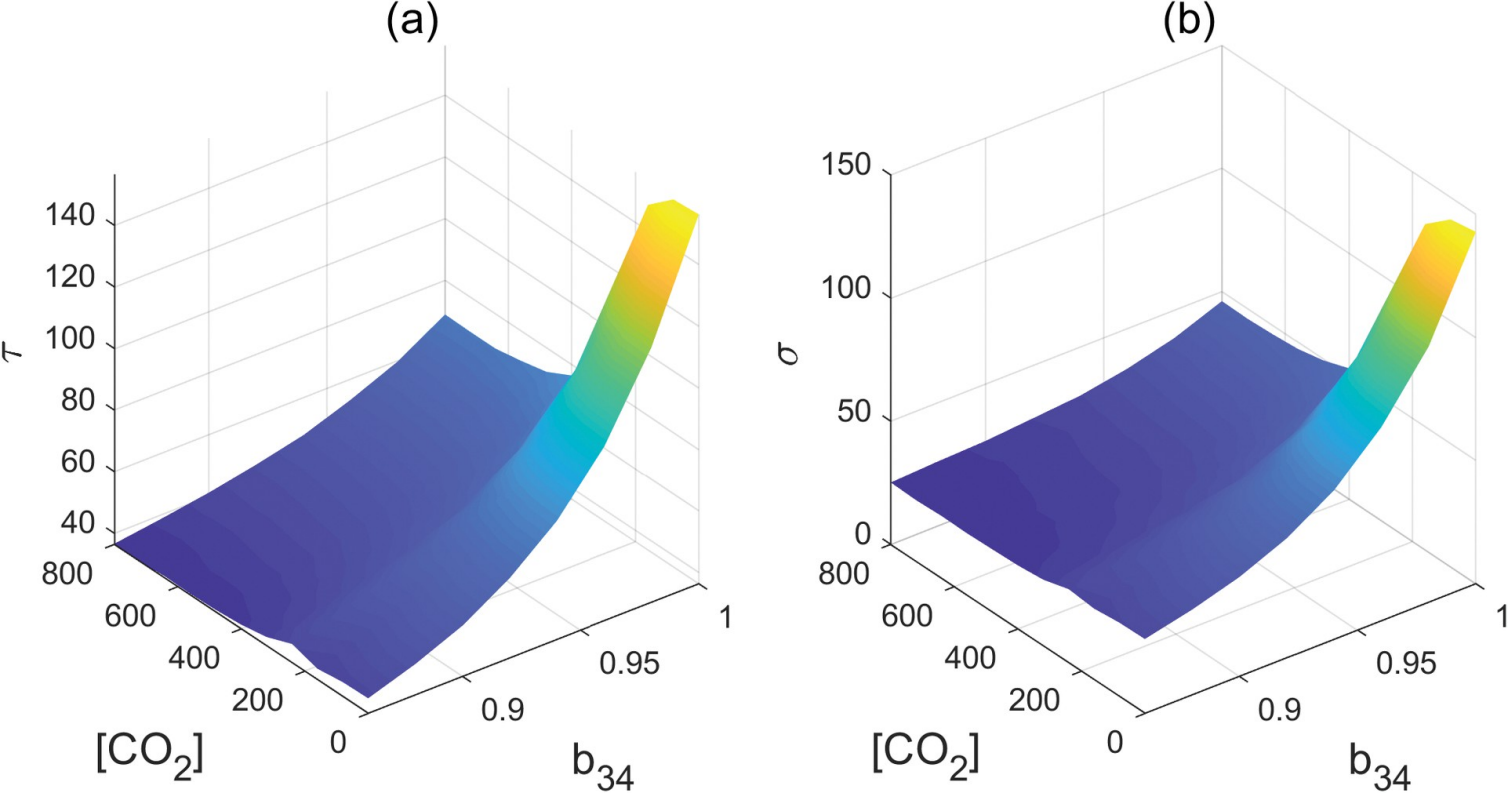
(a) Mean first passage time with different parameter b_34_ and [CO_2_] (b) Standard deviation with different parameter b_34_ and [CO_2_].

To understand the impact of AP2s’ feedback regulation of miR172 on flowering time, we explored the MFPT while varying the inhibition-regulated strengths, b_34_, within the range of 0.85 to 1. This exploration was carried out across a spectrum of CO_2_ concentrations ranging from near zero to 800ppm (Fig 8). Our findings indicated that the MFPT exhibited a gradual increase in response to increasing strength values of b_34_ when the CO_2_ concentration increases from 300 to 800ppm. However, the MFPT displayed a steeper decline when CO_2_ concentrations were below 300ppm.This suggests that the negative feedback regulation of miR172 by the AP2 family might serve to mitigate the effect of increasing levels of CO_2_ and stabilize flowering time. The variance observed in the FPT distribution (Fig 8B) exhibits a parallel trend to that of the MFPT, contributing to the stabilization of fluctuations.

**Fig 9:**
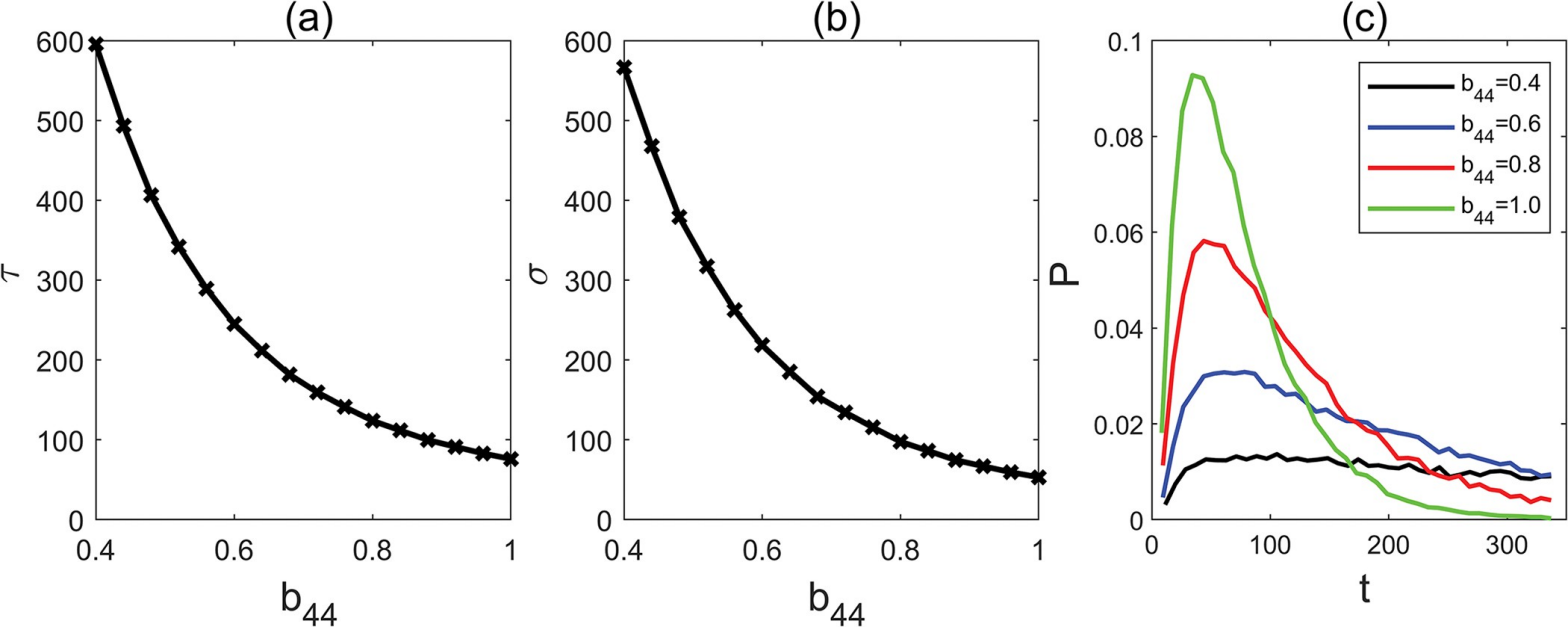
(a) Mean first passage time with different parameter b_44_. (b) Standard deviation with different parameter b_44_. (c) The distribution of first passage time with different parameter b_44_.

Prior experiments have provided evidence that the AP2 family transcription factors have the capacity to bind to the promoters with each other to suppress their expression To investigate this regulatory mechanism, we employed our model to examine the MFPT while varying self-repressor parameter b_44_ (Fig 9A and 9B). We found that the MFPT rapidly decreased as the parameter b_44_ increases. This signifies that an increase in self-repressor strength can greatly accelerate flowering time in response to increasing CO_2_ levels. Conversely, when the expression levels of AP2 family members increase due to a reduction in miR172, we observed a swift increase in MFPT with a decrease in the strength of b_44_, thus greatly delaying flowering time.

We further showed that the distribution of FPT becomes narrower as b_44_ increases (Fig 9C). This observation indicates that an elevated parameter b_44_ can reduce the variability of flowering time. In summary, our results demonstrates that AP2 family, through its suppression effects on miR172 and its own family members, can significantly enhance its output signals while maintaining a delicate balance.

**Fig 10:**
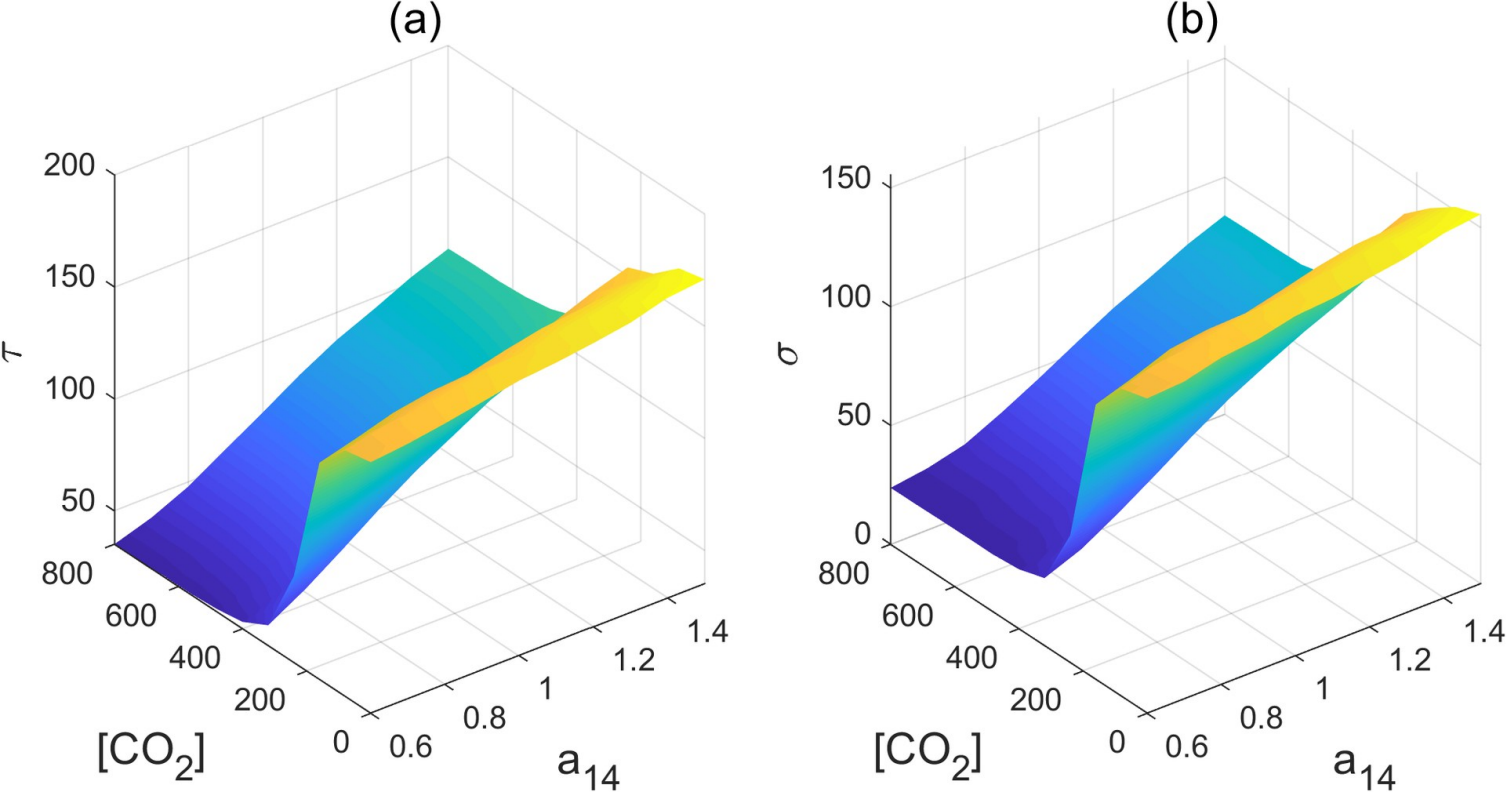
(a) Mean first passage time with different parameter a_14_ and [CO_2_] (b) Standard deviation with different parameter a_14_ and [CO_2_].

AP2 family members can also transcriptionally promote the expression of miR156, as indicated by the a_14_ regulation. Our findings indicate that the effect of a_14_ closely resembles that of the b_34_, both of which delay flowering time and alleviate the impact of increasing CO_2_ concentration (Fig 10). The feedback regulations within the network are required for the stable maintenance of both the juvenile and flowering states.

**Fig 11:**
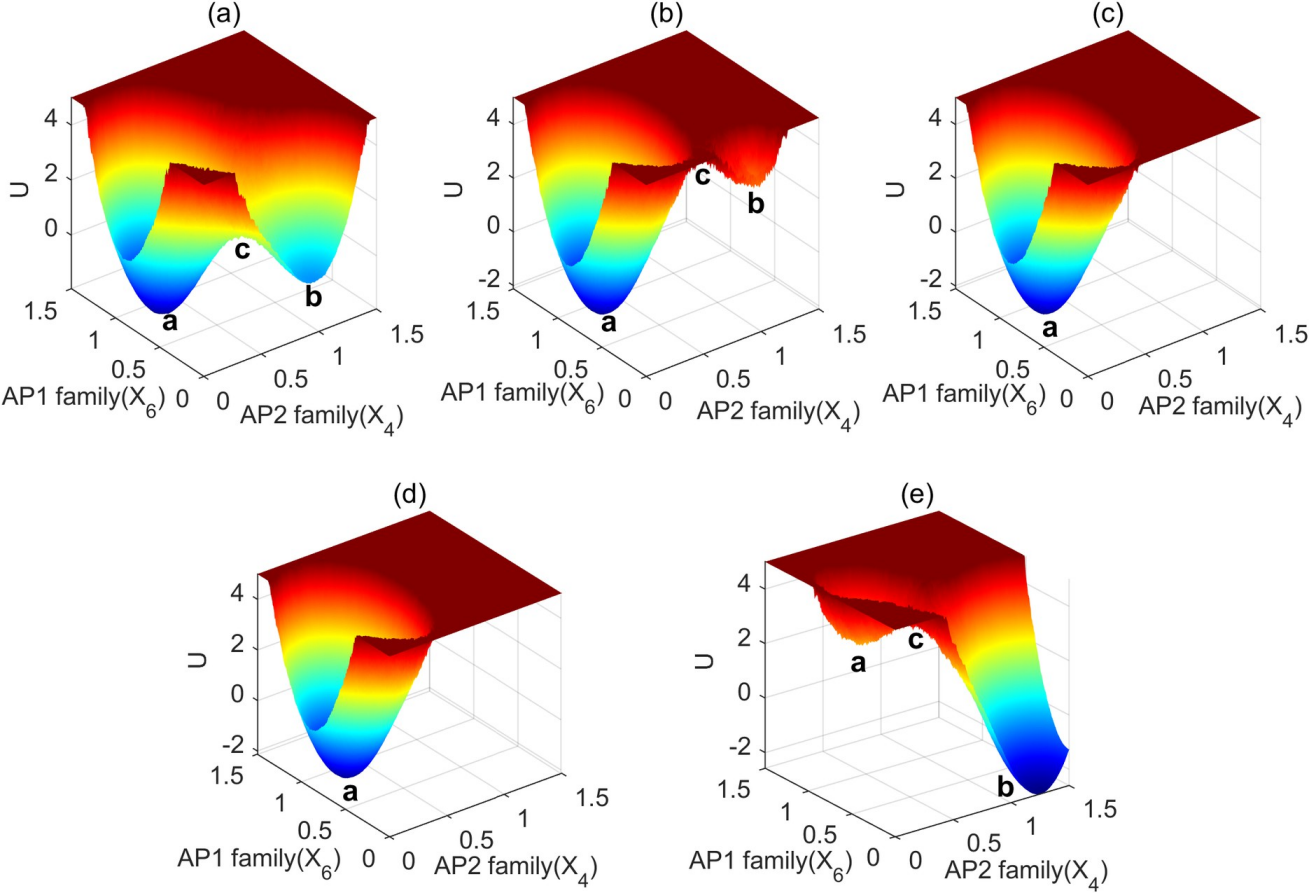
The landscapes of the flowering network after removing different feedback regulation. (a) the normal condition; (b) removeing the SPLs positive feedback regulation of miR156 (a_12_ = 0); (c) removing the AP2 family positive regulation of miR156 (a_14_ = 0); (d) removing the AP2 family negative regulation of miR172 (b_34_ = 0); (e) removing the AP2 family self-suppression (b_44_ = 0).

Given the complexity arising from multiple feedback regulations among the network components, we conducted a study involving the removal of each feedback regulation while observing its consequences on the two steady states, barrier height, and state transition (Fig 11). Under normal conditions, we observed two steady states: the juvenile “b” basin and flowering “a”, with the transition indicated by the barrier height “c”, (Fig 11A). In the absence of a12, which represents SPLs’ positive feedback regulation of miR156, we observed that the juvenile state basin “b” became significantly shallower. This resulted in a highly unstable juvenile state that could rapidly transit to the flowering state (Fig 11B).

In the absence of the “a_14_” representing the AP2 family’s positive regulation of miR156 (Fig 11C), or the absence of “b_34_”, which signifies the AP2 negative regulation of miR172 (Fig 11D), we observed the disappearance of the “b” basin. This signifies the loss of the juvenile state.

In contrast, when the “b_44_” regulation representing the AP2 family’s self-suppression is removed, the plants remain locked in the basin, “b” (Fig 11E). This indicates a prolonged juvenile state with minimal chances of transitioning to the adult state and flowering.

Collectively, our findings demonstrate that all the feedback regulations are essential for maintaining the two steady states, namely the juvenile and flowering states, and for enabling a normal transition between them.

### The miR172-AP2s circuit is the most sensitive regulation within the network

**Fig 12:**
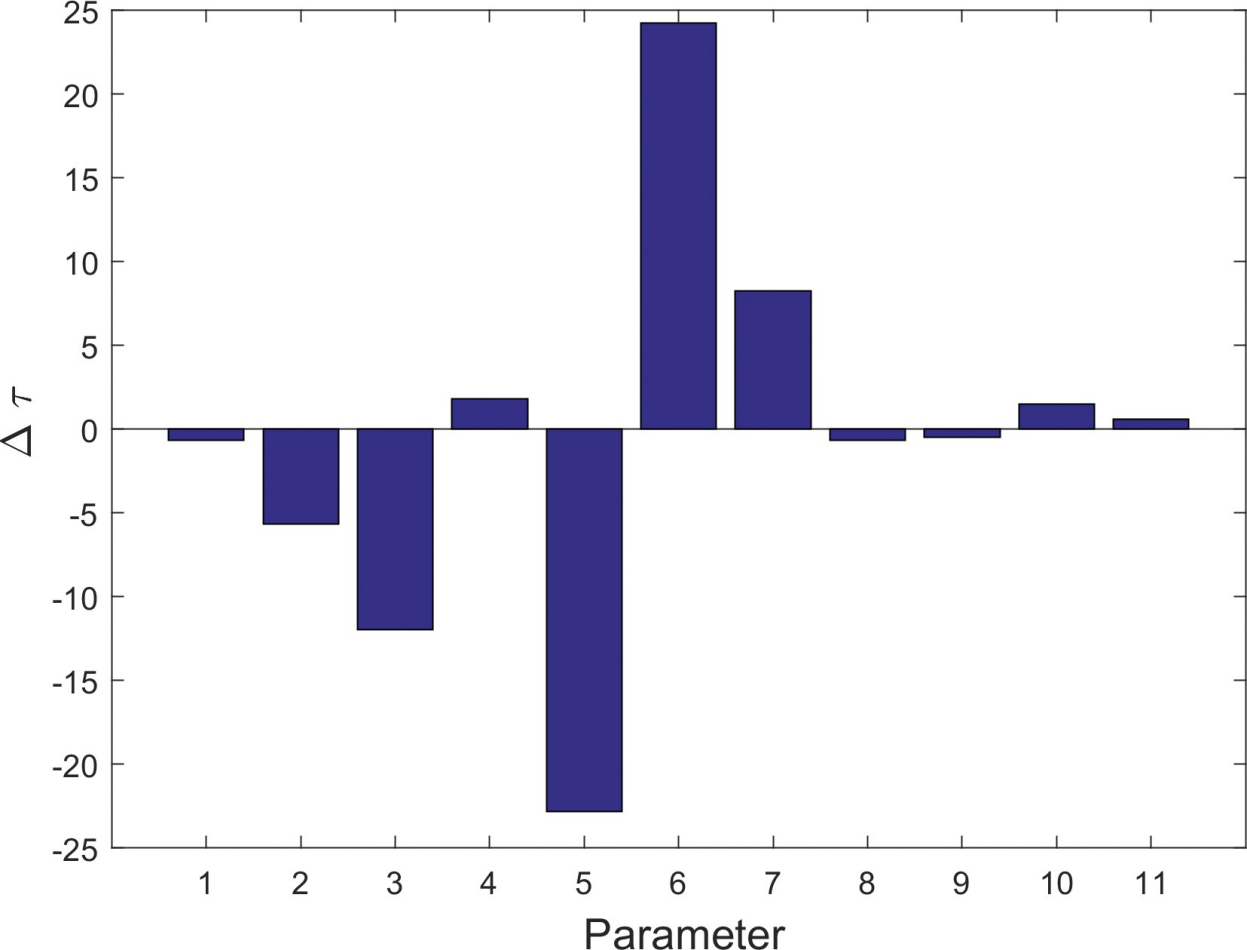
Global sensitivity analysis in terms of the MFPT. (Negative values mean accelerated the flowering time.) (Parameter: 1. a_12_; 2. a_14_; 3. b_21_; 4. a_32_; 5. b_34_; 6. b_43_; 7. b_44_; 8. b_54_; 9. a_65_; 10. [CO_2_]; 11. [SVP]).

To assess the sensitivity of regulatory elements within the network, we conducted a global sensitivity analysis by estimating the change in MFPT with a 5% decrease in each regulation parameter (Fig 12). We found the pivotal roles of two specific steps in modulating flowering time: b_43_, responsible for miR172-mediated suppression of the AP2 family, and b_34,_ governing the feedback suppression of the AP2 family by miR172. Notably, these two factors exhibit the highest sensitivity to alterations. When the strength of miR172-AP2 interaction (b_43_) is reduced by 5%, a significant delay in flowering time is observed. In contrast, a 5% reduction in the feedback suppression of miR172 by the AP2 family, b_34_, results in a substantial acceleration of flowering. Although both a_14_ and b_34_ regulate the network similarly, b_34_ demonstrates significantly greater sensitivity compared to a_14_. Furthermore, a 5% reduction in b_44_, responsible for the self-suppression of the AP2 family, delays flowering time significantly, although this effect is much less pronounced than that observed for b_43_, the feedback suppression of AP2s by miR172.

Sensitivity analysis also reveals that the regulation of miR156 is also sensitive. A 5% decrease in miR156 regulations, either a_14_ or b_21_, results in a significant advancement of flowering times. However, the reduction in miR156 and SPLs regulations (b_21_ and a_12_) can accelerate flowering time, but their impact is not strong due to the positive feedback loop involving SPLs and miR156.

Interestingly, a 5% reduction in b_54_ and a_65_ did not lead to a significant acceleration in flowering time, suggesting that these regulatory elements exhibit robust levels of control.

Our results demonstrate that within the miR156 network, the regulation of miR172 on the AP2 family and the feedback regulation of miR172 by the AP2 family are most sensitive, whereas the miR156-mediated suppression of SPLs and the self-suppression within the AP2 family self-suppressions are also sensitive, albeit to a lesser degree Interestingly, both the SPLs’ regulation of miR156, “a_12_”, and their regulation of miR172, a_32_, are relatively insensitive, requiring a major change in strength to exert a noticeable impact. This finding also extends to other factors, such as s the effect of CO_2_ levels SVP regulation of miR172 (S2A–S2C Fig), and the regulation of floral pathway integrators like FT, as well as floral meristem identity genes such as FUL, all of which exhibit insensitivity, requiring a significant alteration in strength to induce a change in flowering time.

## Discussion

In this study, we investigate the robustness of the miR156-regulated transcriptional network in response to increasing CO_2_ levels. We gained insights into various regulatory elements contributing to this network’s robustness. The miR156 pathway, crucial for regulating developmental flowering time and production in not only *Arabidopsis* but also across a spectrum of plant species, exhibits a remarkable level of conservation from moss to trees [7,12,24,33,34]. Genetic studies have convincingly demonstrated that perturbations in this pathway, achieved through genetic mutations or overexpression can significantly influence plant flowering time and production [6,10,33,35]. It is noteworthy that both biotic and abiotic stressors, including heightened CO_2_ levels and temperature fluctuations, have capacity to modulate the expression of miR156 and its homologue, miR157 [20]. Prior studies have established a correlation between the expression levels of miR156 and photosynthetic sugar levels, revealing that high sugar levels can inhibit miR156 expression [21,22,23]. Furthermore, it is well-known that elevated CO_2_ levels can enhance the rate of photosynthesis. In fact, our gene expression data analysis indicates an upregulation of certain genes associated with photosynthesis in response to higher CO_2_ levels, which could potentially augment the process of photosynthesis. Therefore, heightened CO_2_ levels lead to an increase in sugar production. This, in turn, can potentially affect the expression of miR156, and subsequently regulate various components downstream of the pathway, ultimately impacting flowering time.

Our study builds upon the miR156 and miR172-regulated flowering time network in *Arabidopsis* [6,10,16,36]. We also investigate the influence of CO_2_ levels on this pathway, as highlighted in reference [20]. In *Arabidopsis*, increasing CO_2_ levels lead to the suppression of miR156/157 expressions, consequently triggering downstream changes of this pathway and resulting in the early onset of flowering time [20]. Our study delves into the dynamic alternations within this network in response to elevated CO_2_, levels, aligning well with these experimental findings.

Drawing from these studies, we explore the entire spectrum of the regulatory factors in the network, including CO_2_ levels, miR156/157, miR172, and the involvement of transcription factors. We analyze how variations in the strength of each regulatory component impact flowering time and its variance. Our findings reveal several noteworthy characteristics: (1) The network displays a high sensitivity to CO_2_ levels via miR156/157 regulation, although with a saturation point. Excessive increases in CO_2_ from 400 to 800ppm fail to induce significant changes in flowering time due to the buffering effect of the miR156/157-SPLs loop, while the network orchestrates dramatically advanced flowering time in response to increasing CO_2_ levels from 200 to 300ppm. (2) Both the miR172-AP2s loop and AP2 family self-suppression mechanisms act as effective modulators of upstream signals, facilitating the transition from the vegetative to the adult phase and flowering initiation. These components exhibit remarkable sensitivity in the network, such that even minor adjustments can lead to significant changes in flowering time. (3) The feedback regulation involving miR156 by AP2s or SPLs also demonstrate sensitivity, although to a lesser extent compared to the miR172-AP2s feedback loop. (4) All feedback regulations are indispensable in maintaining both juvenile and adult states as well as the transition time between them. (5) Notably, any delay in flowering time correlates with an increase in the variation of flowering time, and vice versa. This correlation suggests the presence of an adaptative mechanism within the network, designed to respond to environmental challenges and fluctuations.

This study reveals the remarkable degree of control within the miR156 network, characterized by a multitude of logically interconnected features. It underscores the pivotal role of miRNAs in regulating the transition from the vegetative to the adult phase transition and in modulating flowering time in response to elevated CO_2_ levels.

### The landscape method is both global and quantitative

It is widely recognized that miRNA exhibits remarkable responsiveness to environmental changes, rendering it a pivotal environmental sensor in various organisms [4,37,38]. Furthermore, miRNAs are acknowledged for their predominant role in targeting key transcriptional factors within developmental pathways, thereby buffering developmental variations and enhancing the robustness of developmental process [39,40]. In both animals [41,42] and plants [6,10], miRNAs can also serve as developmental phase transition switches and have been shown to form feedback circuits with transcription factors they target [39,43]. Therefore, it is imperative to quantitively ascertain how these miRNAs circuits control the environmental inputs and the robustness of developmental networks.

The regulation of miRNAs has been modelled using miRNA-based chimeric (MBC) circuits [29]. These models encapsulate a greater array of features and details regarding mutual regulations, leading to more precise estimations of parameters and computation performance. However, it is worth noting that these models are deterministic and do not take into account of the influence of the environment, specifically the stochastic fluctuations.

In the realm of complex system dynamics, researchers frequently resort to employing sets of ordinary differential equations as the conventional way for modeling and analysis. However, the method exclusively delves into the deterministic system devolved of noise, and its scope is confined to analyze local stability by identifying fixed points of the system [44].

To gain insights of the global properties of the system, we need to quantify and access global stability. When a driving force can be expressed as a gradient of a scalar potential, we can characterize the system’s dynamics by gradient dynamics and quantify its global behavior using this scalar potential. Notably, in certain physical and biological systems, this scalar potential is directly derived from the interaction potential energy. While we can analyze the system using the equilibrium statistical mechanics, it is imperative to note that this approach is applicable exclusively to the system in equilibrium. In general, the driving force of a complex nonlinear system does not always conform to a pure gradient of a scalar potential. Consequently, equilibrium statistical mechanics cannot be applied universally. Our landscape and flux theory, on the other hand, explores the system dynamics in the presence of fluctuations and can be used to quantify global stability in general systems. In this study, we harnessed landscape quantifications to investigate the dynamics of the miR156/157 network and its relationship with flowering time.

### The miR156-SPLs circuit motif serves as a buffer for the environmental inputs to the network

It has been shown that miR156 targets the members of the SPL transcription factor family, resulting in cleavage of their respective mRNAs. Additionally, SPLs can transcriptionally upregulate the expression of miR156 precursor. This feedback loop presumably establishes a regulatory boundary for the levels of both miR56 and SPLs. Indeed, our research findings support the notion that the miR156-SPLs feedback loop mitigates changes in flowering time to some extent while exhibiting minor variability (Fig 6). Our sensitivity analysis reveals that both the promotion of miR156 by downstream AP2 transcription factor family members and the downregulation of SPLs by miR156 are highly sensitive; a mere 5% reduction in either can significantly alter flowering time (Fig 12).

Similar feedback regulatory mechanisms have also been found in other species. For example, in human hematopoietic cells, there exists a feedback loop between C-myb and miR-15a, and in Glioblastoma cells, miR-21 targets p53 to reduce signal noise [45]. In *C*. *elegans*, a feedback circuit involving let-7-family miRNAs and DAF-12 has been demonstrated to integrate environmental signals into developmental timing, affecting the transition from L2-to-L3 larvae stages or L2-to-dauer stages [41]. Furthermore, miRNAs have been shown to function in bi-directional signal transduction processes, sensing changes in the extracellular environment and modulating cell behavior accordingly [46].

Thus, our data strongly suggest that the miR156-SPLs circuit motif is necessary for plants to buffer the impact of CO_2_ on developmental flowering time and is highly sensitive to even minor perturbations.

### The miR172 -AP2 family circuit motif can amplify the miR172 signal and is the most sensitive to perturbations

This circuit, involving miR172 and AP2s, exhibits a high degree of conservation across plant species. In normal developmental processes, miR172 downregulates the expression of all AP2 family members, including TOE2, SNZ, SMZ, TOE1, and TOE3 and AP2 itself. This downregulation occurs primarily through the suppressing of their translation, with minor cleavages of their mRNAs resulting in minimal changes in mRNAs level. However, under stressful conditions such as elevated temperature and increased CO_2_ levels [20], the miRNA levels of AP2s family members undergo significant alterations.

Two feedback mechanisms contribute to the behavior of the miR172-AP2s circuit. First, AP2s can transcriptionally suppress the expression of miR172 precursors, thus, amplifying the miR172 signal. Secondly, a genome-wide ChIP-chip binding study has revealed that SMZ, one of AP2 family members targeted by miR172, binds to all other five AP2 members [17]. Similarly, AP2 itself has been found to bind to all other five AP2 members [16], suggesting a suppressive regulatory network within the family. This regulatory interplay may limit the extent of the suppression of the AP2 family by miR172.

Our quantitative investigations align with prior experimental findings, which has shown that overexpressing miR172 in plants results in an early-flowering phenotype [5,47], while overexpressing AP2s delays flowering times [17]. Additionally, we provide evidence show that the miR172 signal can be modulated through both suppressive feedback mechanisms: feedback from the AP2 family and the self-suppression of AP2 family members. The inhibition of AP2 signals activates the flower pathway integrators, SOC1, FT, and LFY, which in turn activate flower meristem identity genes, AP1, FUL, CAL, and LFY, resulting in the flowering time change.

Our sensitivity analysis suggests that the miR172-AP2s circuit motif plays a decisive role in controlling plant flowering time. Even a minor increase of 5% in the strength of parameter b_43_ (the suppression of AP2s from miR172) or b_44_ (self-repression of AP2S), can significantly accelerate flowering time while narrowing the range of variation, while a mere 5% increase in parameter b_34_ (representing the suppressive regulation of miR172 by AP2s), can significantly delay flowering time (Fig 12). This finding might provide an explanation for the necessity of maintaining stable miR172-regulated mRNA levels to prevent drastic changes in flowering time during development [48,49]. Moreover, our studies demonstrate that increasing levels of CO_2_ can reduce AP2s, resulting in an early flowering time change.

Our results demonstrate that the suppressive feedback interactions involving miR172 and AP2 family members, as well as among AP2 family members themselves represent the most sensitive feature within the network responsible for modulating the miR172 signal.

### All of these feedback regulations are indispensable

Our data reveals that the removal of SPLs’ positive feedback regulation of miR156, the elimination of the AP2 family’s positive regulation of miR156, or the inhibition of the AP2 family’s negative regulation of miR172 can all induce an unstable juvenile state and the transition to flowering. These findings are supported by previous experiments. For instance, SPL9 and SPL10 have been shown to upregulate miR156 expression through positive feedback mechanisms, leading to delayed flowering time [6]. Additionally, AP2 is known to transcriptionally suppress the expression of miR172, creating a feedback loop within the miR172-AP2 interaction [16]. The interplay among AP2 family members also involves negative regulation [16,17]. Our findings not only support these experimental observations, but also underscore the dramatic effects that arise from the removal of these three feedback regulations.

Conversely, when the self-suppression of the AP2 family is removed, it can enhance the expression of AP2s, resulting in a significantly prolonged juvenile state. This observation aligns with experimental evidence indicating that AP2 family members function as suppressors of flowering [17,48].

Our results demonstrate the significance of these feedback regulations, which are indispensable for maintaining both juvenile and adult states and ensuring a smooth transition between them.

### Elevated CO_2_ levels impact plant adaptation by modifying the variability in flowering time

We previously observed that seeds from the same *Arabidopsis* plant resulted in individual plants displaying flowering time from a few days to several weeks [20]. This wide range of flowering time provides an advantage for plant species to adapt to unpredictably aberrant environments. Our results show that delayed flowering time is consistently correlated with a broader range of flowering time, and conversely, accelerated flowering time in response to increasing CO_2_ concentrations may result in a narrower range of flowering times, making plants less adaptable to environmental changes.

### Increasing CO_2_ from 200 to 300ppm greatly advances flowering time

To our surprise, our findings revealed a significant advancement in flowering time when CO_2_ levels increase from 200 to 400 ppm, with the most notable effect occurring between 200 and 300 ppm (Fig 3). It is widely acknowledged that over the past 450,000 years, atmospheric CO_2_ levels have fluctuated between 200 and 280 ppm, accompanied by temperature variations during glacial and interglacial periods [50]. Currently, the earth is in an interglacial period. Our results hint that this intricate network has evolved enabling plants to effectively adapt their flowering times to the fluctuations in CO_2_. This raises the question of whether this robust mechanism we identified could also prove highly effective in adapting to the expected rise of CO_2_ levels from 400 to 800 ppm due to human activities. Further experiments may yield valuable insights into addressing this question.

In this study, we conducted quantitative investigation of the miR156 network, determined the sensitivities of its regulatory features, and predicted the timing of plant flowering in response to increasing levels of CO_2_. We outlined how various components and circuits in the network affected its robustness and highlighted how two circuit motifs regulated by miR156 and miR172. The formal plays a crucial role in buffering environmental input signals, while the latter is integral to a plant’s commitment to transitioning from the vegetative to the adult phase and initiating the flowering process. Our results shed lights on the dynamic behavior of the network, provided insights into the interpretation of previous experimental data, and indicated evolutionary advantages conferred by miRNAs-regulated feedback loops enhancing plant adaptation. Our study established a physical base for understanding how plants modulate their flowering time in response to increasing levels of CO_2_ and for developing genetic engineering strategies to manipulate flowering time, ultimately contributing to improved plant productivity and ecosystem stability.

Over 60 studies have demonstrated that increasing CO_2_ can either accelerate or delay flowering time in various plant species and crops [51]. Considering the high conservation of the miR156/157 network, our current research approaches could be adapted to investigate how CO_2_ affect flowering time in other plant species. Additionally, our studies may provide insights into predicting the impact of other environmental factors such as temperature, humidity, and drought on flowering time, as these factors can affect photosynthetic sugar production and the function of this network.

## Supporting information

S1 Text Extended the section of Result. (DOCX)

S1 Fig (a) Mean first passage time with different parameter a_32_. (b) Standard deviation with different parameter a_32_. (c) The distribution of first passage time with different parameter a_32_.
(TIF)

S2 Fig (a) Mean first passage time with different parameter [SVP]. (b) Standard deviation with different parameter [SVP]. (c) The distribution of first passage time with different parameter [SVP].
(TIF)

S1 Code The relevant C code. (C)
