# Supplementary material for: High CO2 adaptation mechanisms revealed in the miR156-regulated flowering time pathway
Source: PLoS Comput Biol. 2023 Dec 20;19(12):e1011738. doi: 10.1371/journal.pcbi.1011738 (PMC10775972; doi:10.1371/journal.pcbi.1011738)
Supplement: S1 Text — (DOCX) [file pcbi.1011738.s001.docx]

Supplementary Materials for

**High CO_2_ adaptation mechanisms revealed in the miR156-regulated flowering time pathway**

Kun Zhang, Erkang Wang, Qiong Alison Liu*, Jin Wang*

*corresponding author: Qiong Alison Liu: alisonliu33@yahoo.com

*corresponding author: Jin Wang: [jin.wang.1@stonybrook.edu](mailto:jin.wang.1@stonybrook.edu)

The value of parameters is as follows for the model of the miR156/157 regulatory network

| **Parameters** | a_12_ | a_14_ | b_21_ | a_32_ | b_34_ | b_43_ | b_44_ | b_54_ | a_65_ | [SVP] | a |
| --- | --- | --- | --- | --- | --- | --- | --- | --- | --- | --- | --- |
| **Value** | 1 | 1 | 1 | 1 | 1 | 1 | 1 | 1 | 1 | 1 | 0.1 |
| **Parameters** | b | bm | bm_21_ | bm_34_ | bm_43_ | bm_44_ | bm_54_ | n | S | k | c |
| **Value** | 1 | 1 | 1 | 1 | 1 | 0.3 | 1 | 4 | 0.5 | 1 | 0.0025 |

Table A. The value of the parameters.

**Increasing strength of the SPLs-miR172 regulation can greatly accelerate plant flowering time**

To understand how the transcriptional regulation of miR172 is controlled by SPLs, we examined the MFPT with continuous alterations in the parameter a_32,_ which stands for the strength of SPLs regulation of miR172a, b (S1 Fig (a)(b)). We found that with an increase in a_32,_ the MFPT decreases monotonically, and the distribution of the FPT becomes narrower (S1 Fig (c)). This suggests that increasing the strength of SPLs regulation of miR172 can greatly accelerate flowering time and reduce the flowering time range.

Similarly, the regulation of miR172 precursored by SVP transcription factor showed the similar behavior as the regulation of miR172 by SPLs (S2 Fig)

To determine how the promotion of AP2 family members for the miR156 expression influences the flowering time, we examined MFPT under different activation strengths a_14_ against different levels of CO_2_ (Fig.10). We found that the MFPT increases monotonically with respect to the increase of the strengths of a_14_ when the CO_2_ concentration is fixed. This means that an increase in a_14_ can delay the mean flowering time under the specific input of CO_2_. However, we also found that the range of the MFPT with the high strength of a_14_ is smaller than that with the low strength of a_14_ when increasing the concentrations of CO_2_. Thus, this miR156 promotion activity by AP2 family counteracts with the miR156 reduction activity by increasing the levels of CO_2_ to stabilize the flowering time. On the other hand, the Standard deviation of FPT distribution (Fig. 6(b)) has the similar stable trend, suggesting that the promotion of miR156 by AP2 family can stabilize the flowering time.
