# Supplementary figures and images for: High CO2 adaptation mechanisms revealed in the miR156-regulated flowering time pathway
Source: PLoS Comput Biol. 2023 Dec 20;19(12):e1011738. doi: 10.1371/journal.pcbi.1011738 (PMC10775972; doi:10.1371/journal.pcbi.1011738)

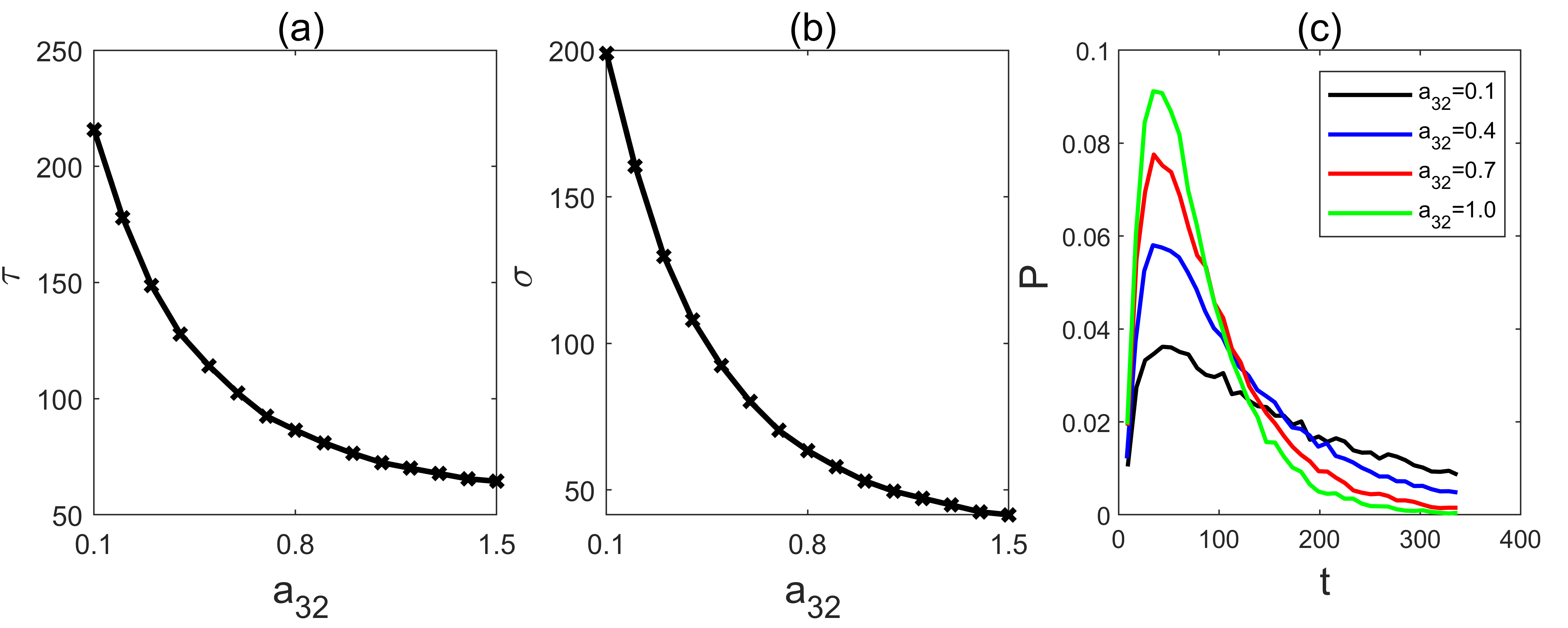

Supplement: S1 Fig — (a) Mean first passage time with different parameter a32. (b) Standard deviation with different parameter a32. (c) The distribution of first passage time with different parameter a32. (TIF) [file pcbi.1011738.s002.tif]

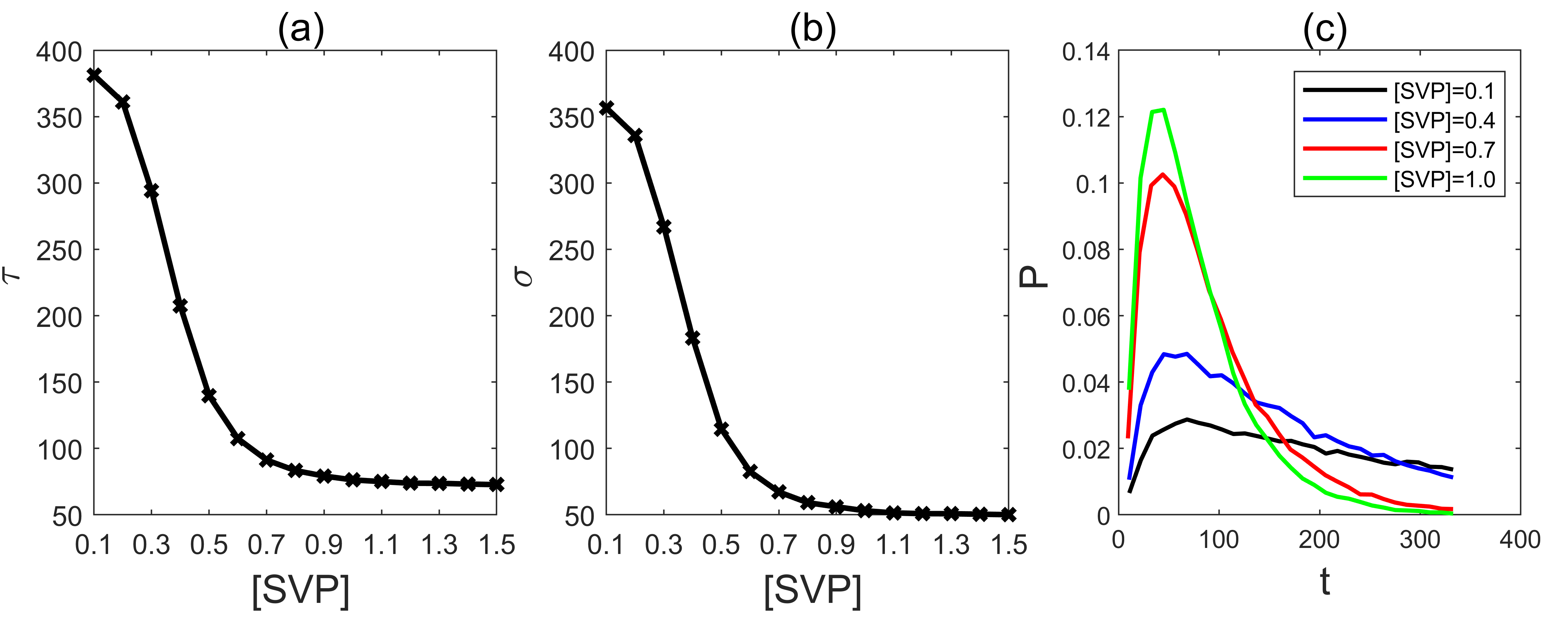

Supplement: S2 Fig — (a) Mean first passage time with different parameter [SVP]. (b) Standard deviation with different parameter [SVP]. (c) The distribution of first passage time with different parameter [SVP]. (TIF) [file pcbi.1011738.s003.tif]
